# Clinicopathological and prognostic characterization of oral lichenoid disease and its main subtypes: A series of 384 cases

**DOI:** 10.4317/medoral.23576

**Published:** 2020-05-10

**Authors:** José Manuel Aguirre-Urizar, Javier Alberdi-Navarro, Irene Lafuente-Ibáñez de Mendoza, Xabier Marichalar-Mendia, Begoña Martínez-Revilla, Carmen Parra-Pérez, Andoni De Juan-Galíndez, María Ángeles Echebarria-Goicouria

**Affiliations:** 1 Oral Medicine and Oral and Maxillofacial Pathology Units. Dental Clinic Service. Master of Oral Pathology. Department of Stomatology II. University of the Basque Country (UPV/EHU). Leioa, Spain

## Abstract

**Background:**

To clinicopathologically characterize the diagnosis of oral lichenoid disease (OLD) and its main subtypes: oral lichen planus (OLP) and oral lichenoid lesion (OLL), in order to correctly asses their prognosis.

**Material and Methods:**

Ambispective cohort study of 384 patients with diagnosis of OLD, based on pre-established clinical and histopathological criteria. We have analysed 272 (70.8%) women and 112 (29.2%), whose mean age was 57.1+/-11.8 years (range 21-90); minimum follow-up time was 36 months. A specific protocol was designed for this study, where we gathered the data of each patient, including malignant transformation.

**Results:**

OLP was diagnosed in 229 cases (77.9%) and OLL in 85 (22.1%). Tobacco consumption was found in 20.3% of the patients and alcohol intake in 41.1%. Liver pathology was present in 10.7% of the cases, thyroid pathology in 11.5%, arterial hypertension in 15.6%, diabetes mellitus in 7.6%, psycho-emotional disorders in 33.3%, skin involvement in 12% and genital involvement in 4.9%. Ten patients (2.6%) developed an oral squamous cell carcinoma, 5 (1.7%) with OLP and 5 (5.9%) with OLL.

**Conclusions:**

OLD is a potentially malignant disorder of the oral mucosa which has to be correctly diagnosed as either OLP or OLL, since the risk of malignancy of these subtypes is significantly different.

** Key words:**Oral lichenoid disease, oral lichen planus, oral lichenoid lesion, diagnosis, malignant transformation, prognosis.

## Introduction

Traditionally, the term “oral lichen planus” has been used to name a heterogeneous group of disorders of the oral mucosa, occasionally confusing, whose main nexus was the presence of white papular lesions in the oral cavity that could not be scratched off (1-3). This pathology has triggered multiple controversies regarding its etiopathogenesis, diagnosis, classification, treatment and prognosis. These mismatches have put into question whether it can truly be considered as an oral premalignant disorder (2,4-7).

With the aim of ameliorating the diagnosis and reducing the existing controversy, ten years ago we proposed to gather all of these disorders under the name of “oral lichenoid disease” (OLD) for the first time (8). WHO’s diagnostic proposal (9) had previously been questioned due to its great variability, and above all, the existence of different subtypes of this disorder with distinct prognosis had already been recognised (10-12).

**Figure 1:**
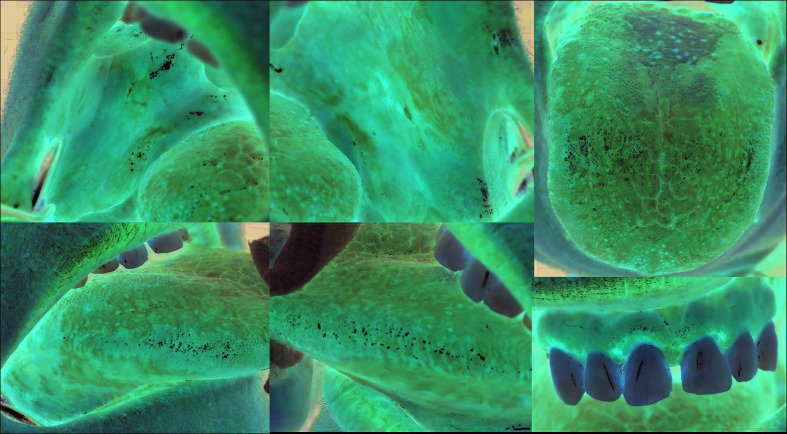
Presence of multiple white papular lesions with reticular arrangement and atrophic-erythematous, erosive-ulcerative and plaque lesions. Bilateral and symmetric distribution. Clinical diagnosis of Oral Lichen Planus.

**Figure 2:**
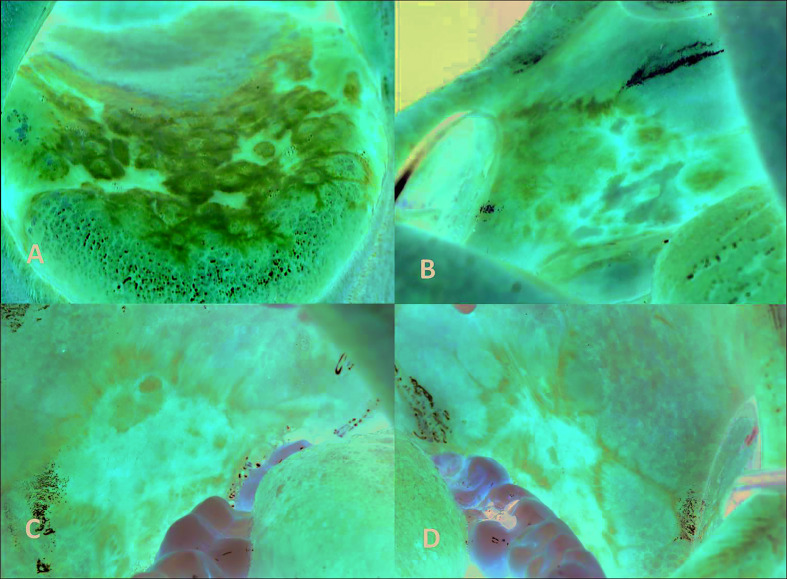
A) Presence of papular, atrophic-erythematous and plaque lesions located only on the tongue. Clinical diagnosis of Oral Lichenoid Lesion. B) Presence of papular, atrophic-erythematous and ulcerative lesions in the right buccal mucosa after treatment with antihypertensive medication. Clinical diagnosis of Oral Lichenoid Lesion. C and D) Presence of papular, atrophic- erythematous and ulcerative lesions in the right and left buccal mucosa in a patient undergoing bone marrow transplantation due to multiple myeloma (graft versus host disease). Clinical diagnosis of Oral Lichenoid Lesion.

**Figure 3:**
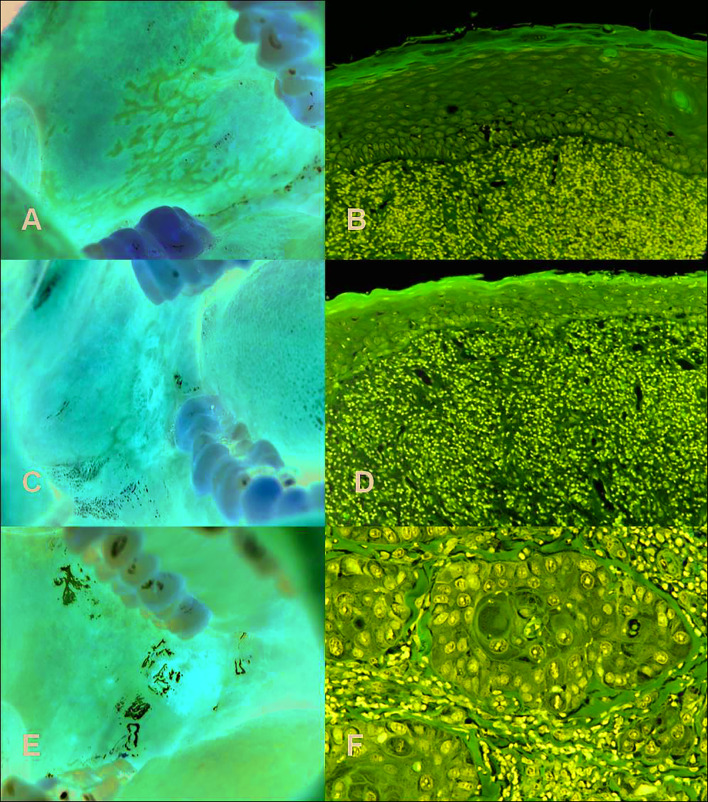
A) White papular lesions with reticular disposition, fundamental for the diagnosis of Oral Lichenoid Disease. B) "Compatible” histopathology in an OLD biopsy (H&E x20). C) Papular and atrophic lesion in the buccal mucosa. D) "Compatible" histopathology of case C with epithelial atrophy, hyperkeratosis and chronic mucositis (H&E x20). E) Erythroplastic lesion of Case C 4 years after the diagnosis of OLD. F) Histopathology of case E showing a squamous cell carcinoma (H&E x100).

The “white papule” is the characteristic diagnostic clinical lesion of OLD, usually “linear” and with a “reticular” arrangement, that must always be present in order to make its clinical diagnosis (8) (Fig. 1, Fig. 2, Fig. 3).

The two main clinical subtypes of OLD are: “oral lichen planus” (OLP) and “oral lichenoid lesion” (OLL). OLP characteristically shows a clinical pattern with multiple lesions, bilateral and in most cases, symmetric (Fig. 1). In contrast, OLL does not show this pattern and/or exists a “recognizable causal factor” (contact with silver amalgam, drugs, host-versus-graft disease, etc.) (Fig. 2). Removal or resolution of this factor should obliterate the lichenoid disorder. In many cases we are not capable of discovering the causal factor (8).

In all cases clinically compatible with OLD it is necessary to make a histopathological analysis through one or more biopsies in order to: complete the diagnosis, dismiss other possible diseases and assess the presence of epithelial dysplasia (13). We have recognized that there is no pathognomonic histopathological pattern for OLD, nor specific data that would allow us to distinguish its main subtypes and that the histopathology is related to the clinical aspects of the disorder (14,15). On the contrary to what other authors state (10,15), we believe that the presence of epithelial dysplasia does not invalid the diagnosis of OLD (14).

Currently, most authors (5,7,16-19) recognize OLD as an oral potentially malignant disorder, with a low risk of malignancy. Different studies (7,11,12,17,19) have recognised that the prognosis of OLD depends on the subtype in question, with OLL having a higher risk of malignant transformation.

Taking these postulates as a starting point, we have projected this study aiming to characterize the diagnosis of this frequent disease of the oral mucosa and its main subtypes in a series of cases in our environment, in order to simplify and improve its diagnosis and be able to correctly asses its prognosis.

## Material and Methods

We have performed an ambispective cohort study on 384 patients with a clinicopathological diagnosis of oral lichenoid disorder (OLD) at the Oral Medicine Unit of the Dental Clinic Service of the University of the Basque Country (UPV/EHU).

Sample size was 272 (70.83%) women and 112 (29.17%) men, whose mean age was 57.15 years (SD: 11.88), with ages between 21 and 90 years.

The inclusion criteria applied were: 1) patients with diagnosis of OLD (OLP or OLL), following established clinicopathological criteria and 2) patients with at least 36 months of follow-up time. The exclusion criteria applied were: 1) patients with clinical and/or histopathological characteristics of another oral mucosa disease different from OLD and 2) patients whose follow-up time was less than 36 months.

In all cases a specific protocol designed for this study was filled in, where all initial and evolutive data of each patient was gathered. The clinical and histopathological criteria used for the diagnosis of OLD, OLP and OLL appear in Table 1. Patients with clinical diagnosis of OLD whose biopsies showed epithelial dysplasia in the histopathological analysis were not excluded from the study.

With the obtained data, a descriptive and comparative statistical analysis was performed with IBM SPSS v22.0. In the comparative analysis the Chi-squared test was carried out through contingency Tables. Odds ratio (OR) was calculated to assess the risk, with a 95% confidence interval.

## Results

After applying the proposed diagnostic criteria (Table 1), we classified 299 cases (77.9%) as OLP and 85 (22.1%) as OLL.

Mean follow-up time of OLD patients was 71.73 months (SD 43.34), with a range between 36 and 229 months.

Clinical data of the cases of OLD and the subtypes OLP and OLL are shown in Table 2.

We have not recognized any differences among these subtypes regarding gender and age. Nonetheless, the mean age of patients of the OLL group was slightly smaller and showed a bigger number of male patients than in the OLP group.

**Table 1:**
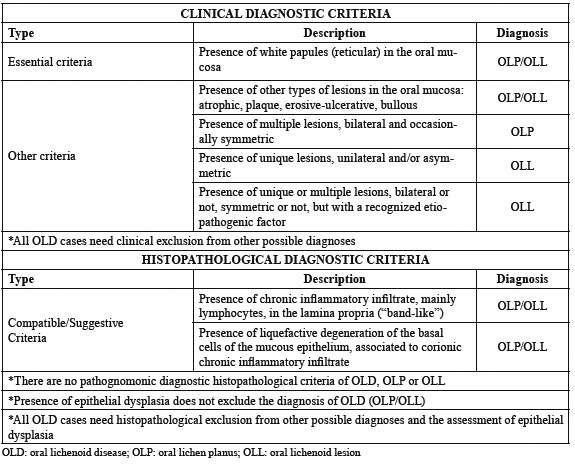
Diagnostic criteria of oral lichenoid disease (OLD) and its subtypes, oral lichen planus (OLP) and oral lichenoid lesion (OLL).

**Table 2:**
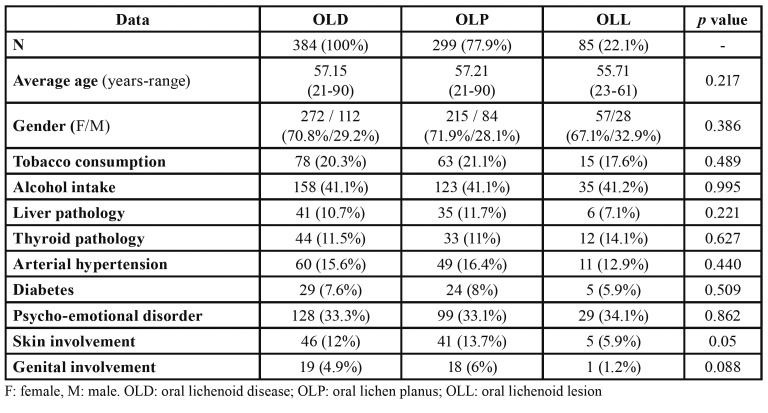
Main characteristics of the patients with OLD included in the study and subgroups OLP and OLL.

We did not observe significant differences between groups regarding tobacco and alcohol consumption, however, the number of patients consuming tobacco was slightly bigger in the OLP group.

Coexistence of liver and/or thyroid pathology at the time of diagnosis of OLD was low. Although no significant differences were observed, patients with OLL presented a lower percentage of liver pathology and a higher of thyroid disease, which mostly corresponded to hypothyroidism. Presence of arterial hypertension or diabetes mellitus in patients was not significant, neither in the total nor in the clinical subtypes.

More than a third part of patients with diagnosis of OLD, in both OLP and OLL subtypes, suffered some kind of psycho-emotional disorder, treated with drugs, mainly anxiolytics or antidepressants.

Cutaneous lesions were present in a small percentage of patients (12%) at the time of diagnosis of the oral disease, mostly in the OLP subtype (*p*=0.05). Genital mucosa affectation was infrequent (4.9%), which mostly corresponded to OLP diagnosed patients.

All data regarding the type of lesions at the time of diagnosis, their location and the evolution of the disease appear in Table 3.

Since being a essential diagnostic criteria of OLD, 100% of cases showed white papular lesions in the oral mucosa at the time of diagnosis. Presence of erosive-ulcerative lesions was significantly more common in the OLP subtype in contrast to OLL (*p*=0.04). Plaque lesions were also more frequent in OLP patients. Atrophic lesions, which clinically appeared as erythematous areas, were common in patients with OLD, although we did not identify any significant difference amongst the two subtypes. Bullous lesions were not observed in any case.

The most common location of the lesions was the buccal mucosa, both in OLD and the subtypes, followed by the tongue (borders and dorsum). Gingiva involvement was seen in 50% of patients, adopting in many cases the clinical appearance of “desquamative gingivitis” with mixed lesions: papules, atrophic and erosive. Other locations (palate, lips, or floor of the mouth) were much less frequent.

Few cases showed complete disappearance of the oral lesions, although it was significantly more frequent in the OLL group than in the OLP (*p*=0.027).

Development of symptomatic erosive-ulcerative lesions was seen at least once during follow-up time of the disease in nearly 30% of patients with OLD, being more frequent in OLP group than in OLL.

Ten patients developed malignant transformation into an oral squamous cell carcinoma, 5 with OLP and 5 with OLL. Malignization was significantly higher in patients with OLL, being the risk 4.6 times bigger than for patients with OLP (OR:4.609; IC9% 1,210-17,565).

Clinical and histopathological data of OLD cases with malignant transformation are shown in Table 4. The mean age of these patients at the time of OLD diagnosis was of 57.4 years and were mainly women. Only two patients were tobacco consumers. The mean time between the OLD and carcinoma diagnosis was 51.3 months (4.3 years), and in 6 cases this evolution time was higher than 2 years. The neoplasms were mostly located in the gingiva and the border of the tongue, and two patients developed more than one primary carcinoma non-synchronous. The histopathological study demonstrated the majority of the cases corresponded to well-differentiated squamous cell carcinoma.

**Table 3:**
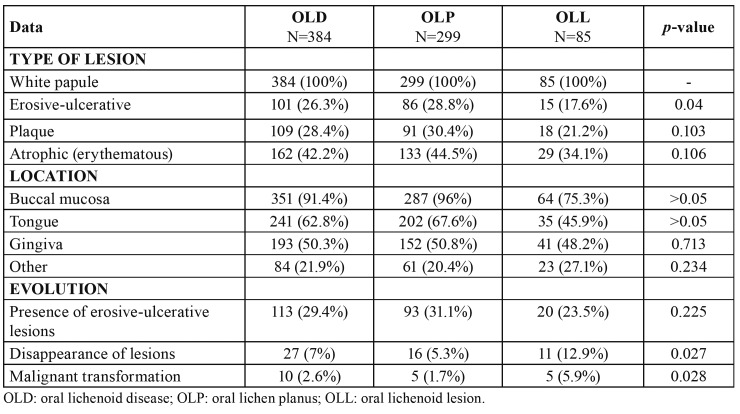
Clinical and evolutive data of the patients with OLD included in the study and subgroups OLP and OLL.

**Table 4:**
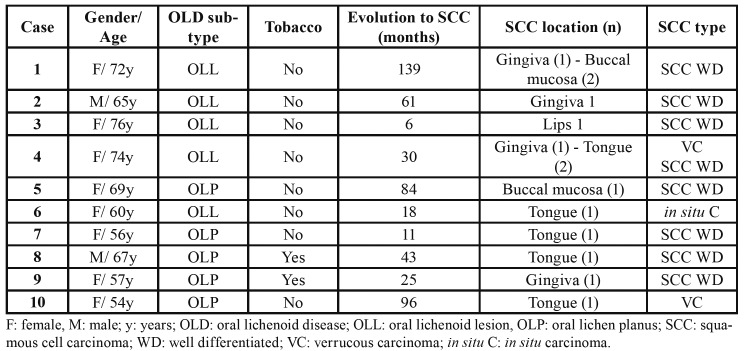
Characteristics of the cases with malignant transformation.

## Discussion

This is the first study to analyse the main clinicopathologically features in a series of OLD cases and those of its main subtypes. Based on the current study, we propose oral lichenoid disease (OLD) to be defined as: a potentially malignant disorder of the oral mucosa that cannot be clinically or histopathologically diagnosed as other specific oral disease and characteristically shows white papules, that are sometimes accompanied by other types of lesions.

In order to diagnose this disease, it is mandatory to perform an exhaustive clinical and histopathological differential diagnosis with multiples pathologies affecting the oral mucosa that also appear with white papules (2,3).

OLD encompasses all the oral mucous disorders previously known as “oral lichen”, “oral lichen planus”, “oral lichenoid lesion”, “oral lichenoid reaction”, “oral lichenoid contact lesion”, etc. (3,18).

We distinguish two main variants of OLD: “oral lichen planus” (OLP) and “oral lichenoid lesion” (OLL). OLP is the most frequent subtype and is characterized clinically by presenting multiple lesions, bilaterally disposed and in many cases, symmetrical (Fig. 1). OLL is less frequent and is characterized by presenting unilateral, unique and asymmetric lesions and/or there is a “recognizable causal factor” (contact with silver amalgam or other products, some drugs, host versus graft disease, etc.), whose elimination or resolution causes the disappearance of oral lichenoid lesions (Fig. 2).

Diagnosis of OLD is fundamentally clinical since, although it is always obligatory to carry out one or several biopsies, there is no pathognomonic histopathological pattern and at most it is only "compatible" or "suggestive", usually as "chronic mucositis" (10,14,15,20) (Fig. 3). Main histopathological suggestive data of OLD are: chronic lymphocytic inflammatory infiltrate with “band-shape” distribution in the lamina propria and liquefactive degeneration of the basal cells of the mucous epithelium (14) (Fig. 3).

Reasons why it is always necessary to make a biopsy in a patient with clinical diagnosis of OLD are: 1) to confirm the clinical diagnosis, 2) to dismiss other specific diseases of the oral mucosa, and 3) to assess the presence of epithelial dysplasia (ED). We consider that the presence of ED in a biopsy of OLD should not invalidate its diagnosis (14). Moreover, currently ED is the gold standard feature to assess the prognosis of oral lesions, including those of OLD (20,21).

OLD is an inflammatory-immunological chronic disorder of the oral mucosa that, in our series, affected mainly adult women older than 50 years (perimenopausal age). This result coincides with the existing traditional knowledge about this disease (1-3,15,16,18).

A controversial data that has been associated with the malignant transformation of this disorder is the presence of smoking habit in oral lichen patients (6,7). In our study tobacco consumption was low in all groups and only two patients who smoked developed an SCC. In contrast, we have recognized a big number of patients that were habitual consumers of alcohol. This result might be related to the diagnostic criteria and the social particularities of the population where each study has been carried out. In our environment a big part of the adult population has a regular tobacco consumption (22). Recent reviews related to the malignant transformation of this disorder (7,17) point out that both the alcohol and tobacco habits ought to be considered as risk factors for malignancy. Thus, we believe it is important to advice all OLD patients to quit smoking and drinking as a general prevention approach.

Just over 10% of our patients with OLD were also diagnosed of liver pathology at the time of diagnosis, which contrasts with some studies from the southern Europe that have even related hepatitis to the pathogenesis and the malignancy of this disorder (3,7,23).

The number of cases with thyroid pathology has reached the 10% in our study and it has been higher in the OLL subtype. These results are similar to those observed in northern Europe studies, where the existence of a specific subgroup of patients with both disorders, OLD and thyroid pathology, has been proposed (24).

A small number of patients in our series showed arterial hypertension or diabetes mellitus at the time of OLD diagnosis. Percentages of these diseases were similar to those identified in adult population from Spain, where 42.6% of people older than 18 years are hypertensive and 13.8% suffer from diabetes mellitus (25). We consider that these results once more emphasise the inexistence of the so called “Grispan syndrome”.

We have discovered that a third part of our OLD patients presented psycho-emotional disorders, mainly anxiety or depression. This association is yet another confusing aspect of OLD that has been investigated for a long time and that could be a simple coincidence in a collective of patients who suffer these disorders (26).

Cutaneous and/or genital involvement in patients with diagnosis of OLD has been rare, and most of the times in the OLP subgroup, which contrasts with what classically has been pointed out (15). Consequently, we consider that OLD is only a “mucocuteanous lichenoid disorder” on a small number of cases, and thus referring to OLD as a “mucococutaneous disease” is not proper. In the majority of the OLD patients their affectation is exclusive of the oral cavity.

As we have already affirmed in the definition of OLD, we believe that the diagnosis of this disease is mainly clinical and that presence of white papules in the oral mucosa must always be found.

OLD is a dynamic disorder that many times shows an irregular clinical course, with “silent periods” (asymptomatic), generally related to the presence of white lesions (papules and plaques) and “active periods”, associated to red lesions (erosive-ulcerative and atrophic). In our study, erosive-ulcerative lesions where more common in the OLP patients. We think that this fact might be linked to systemic antigenic determinants persistent during the onset of OLP, and they would also be associated with the presence of multiple lesions.

White plaques were observed in nearly a third part of the patients and were more frequent in the OLP subgroup. This result would support the new hypothesis of the recent years that some cases of OLD could possibly transform into other entities with multiples white plaques taking place in the oral mucosa like proliferative verrucous leukoplakia (27).

The main locations of OLD have been the buccal mucosa, middle and posterior portions, and the tongue, lateral borders and dorsum, which match with most of the previous studies (1-3). Gingival affectation was also frequent in the shape of “desquamative gingivitis”, that in some cases might be the only active sign of the disease (28).

Because complete disappearance of the lesions is low (<10%), OLD should be generally conceptualized as a chronic oral disorder. As expected, the “disappearance” was significantly higher in the OLL cases. This phenomenon would be justified by the obliteration or healing of the factor which caused the lichenoid process in the first place.

Herein, we prove once again that this mucous disease should be considered an oral potentially malignant disorder that in our study accounted for 2.6% of patients (Fig. 3). This percentage is slightly bigger than those obtained in previous studies from other countries using other diagnostic criteria and different follow-up time: 0.9% (16), 2.1% (10), 1.09% (5), 3.9% (6), 1.1-0.9% (17), 1.14-1.88% (7) and 1.4% (19). Same as other authors (7,15,19) we believe that discrepancy between malignization percentages respond to the variability of diagnostic criteria used in every study, as well as to the little follow-up time in some reports. Thus, it is essential to agree and establish the appropriate and strict diagnostic criteria for this pathology, which will homogenize the studies and reduce the risk of error. This has been one of our fundamental goals and has motivated the diagnostic proposal that we have made (Table 1).

Percentage of malignant transformation was higher in the OLL group than in the OLP, coinciding with previous reports (7,11,12,19). We think that this result on its own justifies the need of a complete clinicopathological diagnostic process in all patients with “white papules” in the oral mucosa, in order to achieve the final diagnosis of either OLP or OLL and establish a correct follow-up and control approach in each case.

Cases of OLD that underwent malignant transformation in our series corresponded, for the most part, to women older than 60 years who did not smoke. These results do not coincide with the usual oral cancer data in our region, where oral cancer normally affects men with a heavy smoking habit (29).

In our patients development of carcinoma occurred after two years since the OLD diagnosis most of the times. Nevertheless, in some cases the time of onset for the malignant lesion took place belatedly, which highlights the need for a good monitoring, by carrying out periodic controls on a continuous basis (6,7).

We have been able to identify the development of more than one primary carcinoma in a patient with OLD. We think this data may be indicative of the cancerous potential of this chronic inflammatory disorder and that, in some cases, a field cancerization may take place (30).

A differential feature regarding the malignization of OLD is that these carcinomas were mostly located in the border of the tongue or the gingiva, and all of them accounted for well-differentiated carcinomas or verrucous carcinomas.

## Conclusions

The main conclusions of our study are: 1) We propose the term “oral lichenoid disease” (OLD) for all the disorders of the oral mucosa that show “white papules” and cannot be clinically or histopathologicaly diagnosed as other oral entities, 2) We believe there are two main subtypes of OLD: “Oral Lichen Planus” (OLP) and “Oral Lichenoid Lesion” (OLL), 3) We propose new clinical and histopathological criteria for the diagnosis of OLD, OLP and OLL and 4) We consider that OLD is an OPMD with a low risk of malignancy (<3%); however, OLL shows a statistically bigger risk for malignant transformation than OLP.

## References

[1] Al-Hashimi I, Schifter M, Lockhart PB, Wray D, Brennan M, Migliorati CA (2007). Oral lichen planus and oral lichenoid lesions: diagnostic and therapeutic considerations. Oral Surg Oral Med Oral Pathol Oral Radiol Endod.

[2] van der Waal I (2009). Oral lichen planus and oral lichenoid lesions; a critical appraisal with emphasis on the diagnostic aspects. Med Oral Patol Oral Cir Bucal.

[3] Carrozzo M, Porter S, Mercadante V, Fedele S (2019). Oral lichen planus: A disease or a spectrum of tissue reactions? Types, causes, diagnostic algorhythms, prognosis, management strategies. Periodontol 2000.

[4] González-Moles MA, Scully C, Gil-Montoya JA (2008). Oral lichen planus: controversies surrounding malignant transformation. Oral Dis.

[5] Fitzpatrick SG, Hirsch SA, Gordon SC (2014). The malignant transformation of oral lichen planus and oral lichenoid lesions: a systematic review. J Am Dent Assoc.

[6] González-Moles MA, Gil-Montoya JA, Ruiz-Avila I, Bravo M (2017). Is oral cancer incidence among patients with oral lichen planus/oral lichenoid lesions underestimated?. J Oral Pathol Med.

[7] González-Moles MA, Ruiz-Ávila I, González-Ruiz L, Ayén Á, Gil-Montoya JA, Ramos-Garcia P (2019). Malignant transformation risk of oral lichen planus: A systematic review and comprehensive meta-analysis. Oral Oncol.

[8] Aguirre Urizar JM (2008). Letter to the editor: Oral lichenoid disease. A new classification proposal. Med Oral Patol Oral Cir Bucal.

[9] Kramer IR, Lucas RB, Pindborg JJ, Sobin LH (1978). Definition of leukoplakia and related lesions: an aid to studies on oral precancer. Oral Surg Oral Mes Oral Pathol.

[10] van der Meij EH, van der Waal I (2003). Lack of clinicopathologic correlation in the diagnosis of oral lichen planus based on the presently available diagnostic criteria and suggestions for modifications. J Oral Pathol Med.

[11] van der Meij EH, Schepman KP, van der Waal I (2003). The possible premalignant character of oral lichen planus and oral lichenoid lesions: a prospective study. Oral Surg Oral Med Oral Pathol Oral Radiol Endod.

[12] van der Meij EH, Mast H, van der Waal I (2007). The possible premalignant character of oral lichen planus and oral lichenoid lesions: a prospective five-year follow-up study of 192 patients. Oral Oncol.

[13] Cortés-Ramírez DA, Gainza-Cirauqui ML, Echebarria-Goicouria MA, Aguirre-Urizar JM (2009). Oral lichenoid disease as a premalignant condition: the controversies and the unknown. Med Oral Patol Oral Cir Bucal.

[14] Alberdi-Navarro J, Marichalar-Mendia X, Lartitegui-Sebastián MJ, Gainza-Cirauqui ML, Echebarria-Goikouria MA, Aguirre-Urizar JM (2017). Histopathological characterization of the oral lichenoid disease subtypes and the relation with the clinical data. Med Oral Patol Oral Cir Bucal.

[15] Cheng YS, Gould A, Kurago Z, Fantasia J, Muller S (2016). Diagnosis of oral lichen planus: a position paper of the American Academy of Oral and Maxillofacial Pathology. Oral Surg Oral Med Oral Pathol Oral Radiol.

[16] Bermejo-Fenoll A, Sánchez-Siles M, López-Jornet P, Camacho-Alonso F, Salazar-Sánchez N (2010). A retrospective clinicopathological study of 550 patients with oral lichen planus in south-eastern Spain. J Oral Pathol Med.

[17] Aghbari SMH, Abushouk AI, Attia A, Elmaraezy A, Menshawy A, Ahmed MS (2017). Malignant transformation of oral lichen planus and oral lichenoid lesions: A meta-analysis of 20095 patient data. Oral Oncol.

[18] Warnakulasuriya S (2018). Clinical features and presentation of oral potentially malignant disorders. Oral Surg Oral Med Oral Pathol Oral Radiol.

[19] Giuliani M, Troiano G, Cordaro M, Corsalini M, Gioco G, Lo Muzio L (2019). Rate of malignant transformation of oral lichen planus: A systematic review. Oral Dis.

[20] Rock LD, Laronde DM, Lin I, Rosin MP, Chan B, Shariati B (2018). Dysplasia should not be ignored in lichenoid mucositis. J Dent Res.

[21] Warnakulasuriya S, Tilakaratne WM, Ranganathan K, Kuriakose MA (2018). Report of a consensus meeting of a group of oral and general pathologists in India on grading of oral epithelial dysplasia. Oral Oncol.

[22] Trofor AC, Papadakis S, Lotrean LM, Radu-Loghin C, Eremia M, Mihaltan F (2019). Knowledge of the health risks of smoking and impact of cigarette warning labels among tobacco users in six European countries: Findings from the EUREST-PLUS ITC Europe Surveys. Tob Induc Dis.

[23] del Olmo JA, Pascual I, Bagán JV, Serra MA, Escudero A, Rodríguez F (2000). Prevalence of hepatitis C virus in patients with lichen planus of the oral cavity and chronic liver disease. Eur J Oral Sci.

[24] Robledo-Sierra J, Landin-Wilhelmsen K, Nyström HF, Mattsson U, Jontell M (2015). Clinical characteristics of patients with concomitant oral lichen planus and thyroid disease. Oral Surg Oral Med Oral Pathol Oral Radiol.

[25] Menéndez E, Delgado E, Fernández-Vega F, Prieto MA, Bordiú E, Calle A (2016). Prevalencia, diagnóstico, tratamiento y control de la hipertensión arterial en España. Resultados del estudio Di@bet.es. Rev Esp Cardiol.

[26] Rojo-Moreno JL, Bagán JV, Rojo-Moreno J, Donat JS, Milián MA, Jiménez Y (1998). Psychologic factors and oral lichen planus. A psychometric evaluation of 100 cases. Oral Surg Oral Med Oral Pathol Oral Radiol Endod.

[27] García-Pola MJ, Llorente-Pendás S, González-Garcia M, García-Martín JM (2016). The development of proliferative verrucous leukoplakia in oral lichen planus. A preliminary study. Med Oral Patol Oral Cir Bucal.

[28] Camacho-Alonso F, López-Jornet P, Bermejo-Fenoll A (2007). Gingival involvement of oral lichen planus. J Periodontol.

[29] Izarzugaza MI, Esparza H, Aguirre JM (2001). Epidemiological aspects of oral and pharyngeal cancers in the Basque Country. J Oral Pathol Med.

[30] Mignogna MD, Fedele S, Lo Russo L, Mignogna C, de Rosa G, Porter SR (2007). Field cancerization in oral lichen planus. Eur J Surg Oncol.

